# Exploration of barriers and facilitators to household contact tracing of index tuberculosis cases in Anlemo district, Hadiya zone, Southern Ethiopia: Qualitative study

**DOI:** 10.1371/journal.pone.0233358

**Published:** 2020-05-22

**Authors:** Legesse Tesfaye, Yohannes Kebede Lemu, Kasahun Girma Tareke, Mulugeta Chaka, Garumma Tolu Feyissa

**Affiliations:** Department of Health, Behavior and Society, Jimma University, Jimma, Ethiopia; The University of Georgia, UNITED STATES

## Abstract

**Background:**

Tuberculosis [TB] is the second leading cause of death from an infectious disease in the world. Intensifying tuberculosis screening and contact investigation strategy is recommended to ensure early diagnosis among household contacts of TB patients. Studies showed that there is low TB contact tracing. There was limited evidence on barriers and facilitators of household contact tracing. Therefore, this study was aimed at exploring barriers and facilitators for household contact tracing of index TB cases.

**Methods:**

A descriptive qualitative study was conducted at Anlemo district, Hadiya zone, Ethiopia from March 12-April 9, 2019. Purposive sampling technique was used to recruit study participants. A total of 16 participants were involved in the study which included health extension workers [HEWs], index TB patients, household contacts of TB patients, health center TB focal and district TB coordinator. Data were collected through in-depth interviews using a semi-structured guide, transcribed verbatim and translated into English. Inductive thematic analysis was employed using ATLAS.ti7.1 software and the findings were presented on major themes, categories, and quotations.

**Results:**

This study found low TB contact tracing and investigation, and explored barriers and facilitators such as monitoring and supervision, training of health workers, logistics and infrastructure, waiting time and institutional readiness, referral, feedback and linkage, human resource, charge for some laboratory, transportation, budget, knowledge, commitment and motivation, workload, distance, social support, economic constrain, and stigma and discrimination for household contact tracing of index TB cases under four themes.

**Conclusions:**

From this study, it was understood that there was a gap in addressing all household contacts. Also, the study explored a wide range of possible barriers and facilitators for it. Explored barriers outweigh the facilitators which might have an implication facilitating the dissemination of TB silently within the community. This underscores the importance of taking action to avert those barriers by developing different strategies to increase TB contact tracing. Therefore, health care providers should have to improve the implementation of contact tracing by designing and developing appropriate strategies that should fit the local context.

## Background

Tuberculosis (TB) is a chronic, infectious disease caused by bacteria generally referred to as the Mycobacterium tuberculosis complex [1–3]. Worldwide, it is one public health issue causing ill-health among millions of people each year, becoming the second leading cause of death from infectious diseases. There were an estimated number of 10 million incident TB cases causing an estimated number of 1.3 million deaths; of which 82% death happened in Africa [4]. Ethiopia is also among the High TB Burden Countries (HBCs), which accounted for 80% of all estimated TB cases worldwide, with an annual estimated incidence of 207/100000 populations and death rate of 33 per 100,000 populations [4, 5].

One major condition that facilitates this high number of incidence and prevalence of TB case, especially, in low and middle-income countries is that there low contact tracing of families of index cases to identify active TB cases or latent TB infection contributing to the rampant transmission of TB within the community [4, 5]. Its prevalence among contacts was 1.2% (microbiologically proven TB), 51.5% of latent TB and 3.4% of MDR or XDR TB; in contrast, among high-income settings, there were 28.1% LTB among contacts [6]. A study conducted in Ethiopia also showed that the overall prevalence of pulmonary tuberculosis among adult household contacts was 7.8% [7].

To combat this problem, in middle and high TB-burden settings, where Ethiopia is part of it, active case finding and contact tracing of index TB cases is recommended for household and close contacts of infectious TB cases [5, 8–10]. Currently, Ethiopia adopted the “End TB” strategy is being implemented, aiming to reduce the incidence and deaths from TB disease by 90% and 95%, respectively, in 2035. Systematic screening of contacts (contact tracing) is one key component among the package of interventions carried out to identify contacts of all ages with undiagnosed TB disease among the contacts of an index case and provide preventive therapy for contacts without TB disease who have increased susceptibility to develop active TB disease following recent infection [4, 5]. To achieve this, the Ethiopian government has set approaches to identify TB cases at the community level by HEWs, and health facility level by TB clinic focal persons [1]. Currently, it is on implementation that the health facilities conduct routine TB screening services for household contacts of active TB patients (1), and the HEWs conduct initial ‘symptom’ based TB screening and refers the contacts [1, 10].

Even though this intervention is being on implementation, different studies conducted in Ethiopia showed that there is low contact tracing of index TB cases when compared with the national plan despite the high prevalence of TB [11, 12]. For better implementation of contact tracing, it is critical to explore the barriers and facilitators towards contact tracing of index TB cases. Therefore, this study was aimed at an exploration of potential barriers and facilitator's contact tracing of index TB cases in Anlemo district, Hadiya Zone.

## Methods and materials

### Study approach, setting and period

A descriptive qualitative study was conducted in Anlemo District, Hadiya Zone, Southern Nation and Nationality People’s Representative (SNNPR) from March 12-April 9, 2019. The district is 214 km far from the capital city, Addis Ababa. The total population of the district is 91464 of which 44817(49%) are males and 46647(51%) are females. There are Protestant, Orthodox and Muslim religious followers and also different ethnic groups within the district even if the majority of them are Hadiya in ethnic origin. Hadiyisa is the primarily spoken language. The District has 5 health centers, 27 rural health posts, and one urban health post. All health centers provide TB diagnostic and treatment services, and health posts give community-based TB treatment services. Additionally, there are eight primary clinics, two medium clinics, and three drug stores [13].

### Participant recruitment and study participants

Two TB clinics focal, one district TB coordinator, six HEWs, three index TB patients and four household contacts of active TB patients were purposively recruited from different health center catchments considering their ability to generate rich information on TB contact tracing. For example, HEWs were recruited based on their performance of referring household contacts to the health center and distance from a health center. Index cases were recruited from both intensive and continuous treatment phases, and from both whose household contacts were traced or not traced. Households hold contacts were recruited considering the distance from health centers [which included those from both far and near to health centers] and clients who screened and not screened for tuberculosis. The sample index cases and household contacts also included from both transferred out and transferred in patients. TB focal and district TB coordinator were recruited based on their experience and responsibility on the TB program.

### Data collection instruments and methods

Data were collected through In-depth interviews (IDI) and key informant interviews using a semi-structured guide prepared in English and then translated to local languages (Hadiyegna and Amharic) and back-translated into English by another independent translator. The guide was prepared to address: a) individual and household-related barriers and facilitators, b) Community-related barriers and facilitators, c) health extension worker related barriers and facilitators, d) health worker related barriers and facilitators, e) health system-related barriers and facilitators to household contact tracing of index TB cases.

Additionally, the document review guide was also prepared and used to collect data on the status of contact tracing of index TB cases from HMIS and registration books; which was carried out before any data were collected to understand the local context in the status of contact investigation. Also, it was used to review the presence of guidelines, supervision checklists, reporting formats, and registration tools. The pretest was done with two participants (one HEW and one index TB patient) at another district, which is the neighbor district of the study district.

The interviews were conducted at the participant’s natural settings and rapport was created with each participant. TB clinics focal were interviewed at their office within the health center. HEWs were interviewed at their respective health post. Two index TB patients were interviewed at the health center. One index TB patient and four household contacts were interviewed within their home, and also district TB coordinator interviewed at his office. At the beginning of each in-depth and key informant interview, the modulator [principal researcher] was briefly described the purpose of the study for the participants. The response of each participant was audio-recorded and notes were taken at each interview. The interviews were ranged from 40-64minutes.

### Data analysis

Inductive thematic analysis was employed to analyze data. Data collection and data analysis were performed simultaneously and before conducting the next data collection importantly emerged ideas were identified by listening audio-recorded material and reading field notes. Also, data saturation was ensured through this process. Verbatim transcription was done by listening audio-recorded material and translated from Amharic and Hadiyegna language to English in support of research assistant, and also checked for completeness and consistency. Reading and re-reading of the data were carried out to become familiarize with data. Then, the principal investigator and the research assistant coded the transcription line by line on ATLAS.ti7 starting from the richest data. Inter-coder consistency was checked, the codebook manual was developed, and then, the principal investigator coded the whole data again and again by refining the codebook. Throughout the coding system, code consistency was checked throughout the coding process by reading and re-reading and re-coding. Then, codes were clustered into categories and themes were developed by connecting related categories. Finally, the report was organized based on major themes, categories, and quotations taken from participants.

### Trustworthiness

Different measures were taken to assure the trustworthiness of the research findings. First, we developed and pre-tested the interview guide. Second, data were collected from diversified study participants recruited from different settings that have relevant experience and expertise. Third, a research assistant was employed who has MPH and experience of conducting research, and he was involved in this research during transcription and coding. Fourth, member checking was done at the end of data collection by summarizing major thematic areas raised during the interview. Research teams also reviewed and gave their comment on the report many times.

Fifth, detailed information about the data collection process, categories deriving process, and decisions making process throughout the inquiry were described. The chronological order of the research report and debriefing of the data was checked by classmates. Sixth, the audit trail was also done by an experienced person on qualitative research on health issues to verify the interpretations of the findings. Seventh, to ensure the transferability of the findings, about the study setting, study participants (demography) and the study findings were described in detail. Has a diversified group of population with different religious and ethnic groups. All health centers and Health posts which are functional currently were on providing TB screening and treatment service. Therefore, interested readers of this research report would make their judgments about the relevance of the findings to their situation and setting and apply the findings of this study in their context.

Eighth, confirmability was achieved through the collecting of thick descriptive data, negative case analyses and arranging for a confirmability audit and establishing referential adequacy. Also, the roles of the research team during the research process were described [i.e. reflectivity and bracketing]. The principal investigator is a BSc nurse in his educational background with four years’ experience of working at different health centers as TB and ART focal and other health center departments. As TB clinics focal, he has carried out responsibilities like monitoring TB patients, tracing and investigating contacts of TB patients, counseling TB patients, prescribe a drug, referring patients to higher health facilities and health extension workers and reporting. The researcher also had taken training on TB and MDR TB and also provided training for HEWs. He had not trained in contact investigation but supervised by different supervisors. The principal investigator is from this study setting. This preconception knowledge and skill benefited the principal investigator to focus on important points while conducting this research; not directed him to conduct a huge bias on the study findings. The other research teams have a health background and experience in TB contact tracing and investigation. Regarding their profession, at the current time, GTF has a PhD and the other (YKL, MC, and KGT) have Master’s degrees. All the research teams have experience in conducting and consulting qualitative researches. To minimize interpretation bias, the principal investigator was focused on the idea of participants, recruited different participants from different kebeles of study settings, documents reviewed by peers and research advisors and focused on the context idea of participants. All the research team checked the consistency of the interpretation with the quotations taken study participants besides the audit trail.

### Ethical considerations

This study was conducted upon approval of the Jimma University Ethical Review Committee and permission obtained from the District Health Office. The purpose of the study was clearly described for each study participant. Verbal consent was taken from each participant. Confidentiality of study participants was kept using code instead of identifying them with their name. The participants were involved voluntarily and they were informed of their rights to participate or withdraw from the study at any time. All of the participants were completed the study.

## Result

### Socio-demographic characteristics of participants

A total of 16 interviews were conducted with a diversified group of individuals. The mean age of the participant was 32.4 years. The majority (10) of the participants were females, and protestant religion followers (Table 1).

**Table 1:** Socio-demographic characteristics of study participants involved in the interview in Alemo district, Hadiya zone, SNNPR, 2019.

Variable	Category	n	Variable	Category	n
Age	20–30	8	Residence	Urban	1
	31–50	8		Rural	6
Sex	M	6			
	F	10	Occupation	Farmer	3
Educational status	Unable to read and write	2		Student	1
	Grade1-8	3		Merchant	1
	Grade 9–12	2		Housewife	2
	10+2	6		HEW	6
	Diploma nurse	1		Health worker	3
	Degree (Health Officer)	1	Category of TB treatment	New treatment	6
	Masters (MPH specialist)	1		Re-treatment	1
Religion	Muslim	5	Household Contacts relation with an index case	Mother	2
	Orthodox	1		Father	1
	Protestant	10		Wife	1

The findings of this study were organized based on the following major themes: health system-related, health workers related, index case and household-related, socio-economic and cultural related barriers and facilitators (Table 2). Except for the presence of social support, and the absence of stigma and discrimination among the community members mentioned as facilitators, most of them are barriers but inherently they can be also facilitators. If their absence is a barrier, their full or partial presence can be a facilitator. For example, on the training of the health worker category, the availability of trained manpower is one facilitator but within it, the shortage of trained manpower is considered as a barrier. So, we did not want to make a demarcation between the two (between barriers and facilitators) while presenting them. Therefore, one who reads this article should have to understand and consider this concept.

**Table 2:** Barriers and facilitators of household contact tracing of index tuberculosis cases in Alemo district, Hadiya zone, SNNPR, 2019.

Major themes	Categories
Health system-related barriers and facilitators	Monitoring and supervision
	Training of health workers
	Logistic and infrastructure
	Waiting time and institutional readiness
	Referral, feedback, and linkage of health facility
	Human resource
	Symptom positive and AFB negative case Charge for Laboratory
	Transportation and budget
Health workers related barriers and facilitators	Health workers knowledge on index TB case contact tracing
	Commitment and motivation health workers
	The workload of the health worker
Index cases and Household contacts related barriers and facilitators	Awareness of the need for screening household contacts of TB cases
	The workload of household contacts
	Index case and household contacts commitment and motivation
Socio-economic and cultural related barriers and facilitators	Distance
	Social support
	Economic constraint
	Stigma and Discrimination

Before coming to these barriers and facilitators, the current contact investigation status from data obtained through document review was elaborated below as an introductory part in the section ‘status of household contact investigation of index TB cases’.

### Status of household contact tracing of index TB cases (Table 3)

**Table 3:** Status of household contacts tracing and treatment linkage of index TB cases in Alemo district, Hadiya zone, SNNPR, from July 2018-April 2019.

Indicators	n (%)
Total number of index TB cases registered	34
Total number of index TB cases for whom household contact registration was conducted	9 (26.5%)
Total number of household contacts registered under nine index TB cases	37
Total number of households screened from household contacts registered under the nine index TB cases	16 (43%)
Total number of under-five children household contacts registered under the nine index TB cases	6 (16%)
Total number of under-five children household contacts screened for TB	4
Total number of under-five children household contacts linked to latent TB treatment with isoniazid (INH)	2
Total number of household contacts screened as symptom positive for TB	8 (50)
Total number of household contacts tested positive for TB case from symptom positive household contacts	0

In the context of this study setting, all health posts and health centers were functional and it was observed that all health centers had registration books for contact registration, but there were no registration books at health posts. From the document review, it was observed that 34 index TB cases were registered from July 2018-April 2019. But, registration was done for household contacts 9 (26.5%) index TB cases. Thirty-seven (37) household contacts were registered under 9 index TB cases of which 6 (16.2%) of them were under-five children. From all these registered household contacts, a total of 16 (43%) household contacts were screened for TB of which 4 (25%) of them were under-five children. Eight (50%) of these household contacts were screened as symptom positive but all of them were checked for laboratory and diagnosed as negative for TB. Two of the four under-five children who were screened for TB linked to latent TB treatment with isoniazid (INH).

In addition to this report, study participants also mentioned that contact tracing and investigation for detection of TB was low at this study setting due to not owning the program by stakeholders, and the limited attention is given to contact tracing and investigation.

“*The guideline says that every individual who has contact with an infected person should have been screened for TB*. *But*, *there is still a gap to register and screen all individuals who have contact*.” (29-year-old, male, TB Coordinator)

### Health system-related barriers and facilitators for household contacts tracing of index TB cases

#### Monitoring and supervision

Health workers mentioned that attention was not given to monitor and supervise health centers and health posts on contact tracing regularly due to problems related to lack of commitment among supervisors. Due to, this there is nothing done in identifying obstacles and solutions, monitoring, motivating health workers, and comparing achievement based on the plan to take action on time. A shortage of transportation was also mentioned as one reason mentioned by study participants that hindered conducting monitoring and supervision.

“*There is no regular monitoring of household contact screening for index Tb cases*…*”* (25-year-old, female, TB Focal)

#### Training of health workers

Contact tracing training was given for health workers and health extension workers integrating it with other TB program training. The reasons mentioned by study participants for low performance of contact tracing and investigation were negligence, low health workers’ commitment, shortage of health workers, the workload on HEWs due to the high burden of health extension packages and gaps in monitoring and supervision.

“*I have attended training on contact tracing while I took basic of TB*, *Leprosy and HIV training*…*” (*25-year-old, female, TB Focal)

#### Logistics and infrastructure

Logistics and infrastructures related barriers were also mentioned by study participants which included electricity interruption, shortage of reagents, non- functional microscopy, absence of TB class or mask at the health post level. The reason for the shortage of reagents was a failure to request and re-supplying timely from the District Health Office and health centers, lack of regular monitoring and supervision. They also mentioned that logistics such as contact screening and referral format, registration book for contact screening, quarterly and monthly reporting format and INH were available. But, there were no registration books at health posts.

“*We are facing a great challenge from shortage of electricity power*. *Most of the health centers didn't have TB rooms and even the available ones are also not well ventilated*… *There is a shortage of reagents…*& *In addition to this*, *some microscopes do not make objects [bacilli] visible correctly*.*”* (29-year old, male, TB Coordinator)

#### Waiting time and institutional readiness

Study participants mentioned a lack of institutional readiness or long waiting time at health facilities as problems that affected contact tracing. They mentioned as there was the repeated appointment of contacts, lack of providing service on time [waiting a long time] and lack of punctuality among health workers. The reasons mentioned for these problems were lack of documenting individual demography like age-appropriately on patient’s card, poor documentation, and storage of documents, poor health workers commitment, problem-related with electricity interruption to search computerized documents at card room and absence electricity to computerized at rural health centers, and shortage of reagent.

“*…But there is a long waiting time at the health center due to a high number of clients*. *The past document was not found in the card room on time*. *Sometime the card would be sent to another service unit even if I wait in another room*. *Some health workers do not come on time or work in another class*. *I would wait some hours until they open the door of service*.*”* (30-year-old, male, TB patient)

### Referral, feedback, and linkage of health facility

Study participants mentioned problems related to referral, feedback, and linkage between health facilities that acted as barriers for TB contact tracing. There were communication gaps between the health center and health posts. TB clinics focal did not regularly inform HEWs to screen and refer household contacts from their respective kebele. Health extension workers, in turn, didn’t receive feedback from health centers. The reasons mentioned for these barriers were high turnover and shift to different units, low monitoring and supervision from district health office, and lack of commitment or motivation from health workers.

*“…*..*But there was a gap in giving information of index case and feedback to health extension workers due to this they did not screen and refer household contacts to health center regularly*.*”*(25-year old, female, TB Focal)

#### Human resource

Participants also mentioned that TB Focal was working on different units of a health center and they conduct activities on shift programs. At a time if TB clinic focal was absent or worked at other units, TB clinic service would be covered by another untrained health worker. There was also a shortage of laboratory professionals which resulted in household contacts in order not to get laboratory service on time, or due to this case, they might be referred to other health facilities for acid-fast bacilli (AFB) checkup. In this condition, household contacts might not go to other health facilities for a checkup because of transportation costs.

“*I am would not work here a full time of a day*. *There are occasions when am off at working hours if I assigned on-duty program night time*. *So*, *I could not get some index cases*. *Even at day time*, *there is a shift that I might work at an outpatient clinic or delivery case team since there is a shortage of professionals*.*”* (25-year-old, female, Focal TB)

#### Symptom positive and AFB negative case charge for laboratory

Index case and household contacts involved in this study mentioned that there is laboratory cost they were asked to pay which made community members in order not to come for screening.

“*I know that screening service and examination of TB are given free of charge*. *But*, *I have paid 70 birrs for x-ray and 40 birrs for blood examination*”. *I saw this during my son*. (43-year-old, female, contact of TB patient)

#### Transportation and budget

One barrier for contact tracing and investigation mentioned by study participants was a shortage of transportation and budget to provide regular supportive supervision and monitoring. The budget constraint was also mentioned as a problem for the building of TB class, maintaining and purchasing microscopy.

“*The challenges are like lack of transportation to provide supportive supervision to all kebeles*.*”*(29-year-old, male, TB coordinator)

### Health worker related barriers and facilitators of contact tracing for index TB cases

#### Health workers knowledge on TB contact tracing

Health workers participated in this study has awareness as contacts of smear-positive index cases should have to be screened for TB. In contrast, HEWs did not know or aware. The reasons were poor adherence with guidelines or no refreshment or on job training given to them. TB focal and HEWs have awareness about the symptomatic screening of TB contacts, how to request and refer household contacts for screening, prescribing of INH for symptom negative under-five children and HIV positive patients and know the benefit of contact screening.

‘*Contact tracing has been performed for household contacts of positive TB cases*. *But*, *I don’t know about other forms of TB category*. *I conduct it only for household contacts of pulmonary positive TB cases and then I would refer them to the health center*.*”* (32 years old, female, HEW)

#### Commitment and motivation health workers

This study also found that health workers were not committed or motivated to trace household contacts for investigation, creating awareness and changing attitudes of contacts and index case, educating and counseling index cases and household contacts, and monitoring and supervising of health facilities on time. Some of the reasons mentioned by study participants were absence any payment for health workers for the risk that would face while conducting on TB clinics, the no-comfortable working setting for TB (i.e. absence well-ventilated class or absence of TB class), absence periodic and continuous monitoring and supervision, and workload. Health workers perceive as they were working at risk area since TB transmitted from the client to health workers with these conditions. They mentioned that they fear this due to having of experience that they have observed one former TB focal who was developed and treated for TB.

“*I haven’t seen anyone who is working that much*, *moving and struggling*, *to refer and screen on time*. *There is also negligence among health workers*, *and we are not committed professionals*.*”* (25-year-old, female, TB Focal)

#### The workload of the health worker

Health workers were reported that workload due to working at different health center units in addition to TB clinics was one of a barrier for contact tracing. The reasons were a shortage of health workers who took training on TB; i.e. only one health worker trained on TB per health center.

“*TB focal has been working at another case team of health centers like Outpatient department*, *under-five clinics or emergency department besides of TB unit*.*” (*35 years old, female, HEW)

### Index case and Household contact related barriers and facilitators for contact tracing of index TB cases

#### Awareness of the need for screening household contacts of TB patients

Study participants, especially index cases and household contacts perceive as it is enough if only index case is treated. They perceive that treating index patients would prevent the transmission of TB. Index case and household contacts had awareness of causes, treatment, transmission, and prevention of TB. In contrast, some do not aware of the transmission of TB through contacts of TB patients. They consider that treating index case, opening window and door, separating utensils from index patients was sufficient to prevent transmission of TB. They had awareness of symptoms of TB, especially cough. They considered themselves as healthy and felt as healthy unless there was no symptom like cough, and wait until severely ill. Besides they gave priority for different tasks rather than coming to a health facility and not willing to come for contact tracing and investigation.

“*We have not coughed or not become sickened*. *So for what screening we go*? *If an individual doesn’t have a cough*, *so he/she go for screening for TB*?*” (45year old*, *female*, *household contact of TB patient)*

#### The workload of household contacts

Providing priority for work is one mentioned barrier mentioned by household contacts and index cases participated in this study. They mentioned that if contacts become healthy and they felt as healthy so that they give priority for work. There were also long waiting times at the health facility to return on time for their work.

“*We did not go for contact screening of TB due to we were busy at work*. *We have the burden of work…*..*”*(45-year-old, female, contact of TB patient)

#### Index case and household contacts commitment and motivation

This study found that there was poor commitment and motivation among index and household contacts to take to health extension workers and health facilities. The reasons mentioned for this poor commitment and motivation were lack of awareness about the importance of contact screening and investigation, lack of awareness on TB, and caring for others and family and interest. Their educational statuses, economic status, giving priority to their work, relying on symptoms especially on cough were also mentioned as a barrier for motivation among index case and household contacts.

“*I did not see anyone who brings their household contacts for screening by own*. *Some index cases repeatedly said “I will bring” but they did not bring their household contacts*. *They say always “I am busy*, *I forgot it or nobody has a cough*.*”* (25-year-old, female, TB Focal)

### Socio-economic and cultural related barriers and facilitators for contact tracing of index TB cases

#### Distance

Study participants also mentioned distance from the health center as a barrier for contact tracing.

“*…there is a distance to go health center which takes around 2*:*30–3*:*00 hour on foot*.*”* (50-year-old, male, Contact of TB patients)

#### Social support

This study also found that TB patients and their household contacts get supports like food, transportation cost, praying during sickness, covering their job and taking them to health facilities from their family and neighbors.

“*Community encourage*, *ask improvement daily*, *even some give food money and all thing covered by the family*. *The community encourages going to a health facility*. *They also pray for my sickness*. *They did not allow to do hard work for social issues like during wedding*, *idir*.*”* (30-year-old, male, TB patient)

#### Economic constrain

A shortage of money was mentioned as an obstacle for visiting health facilities for contact investigation of TB due to need of money for transportation, registration at health facilities and for food during referring and for some laboratory charge at a health facility.

“*The obstacles to go for investigation of TB were*, *for example*, *a shortage of money for transportation and food*. *Due shortage of money individual (contacts) did not go health facility*.*” (*30-year-old, male, TB patient)

#### Stigma and discrimination

Study participants mentioned that index case and household contacts were disclosed their status and community members did not discriminate and stigmatize them.

“*Why I was ashamed to tell about my illness*? *I won't be ashamed to tell*. *I heard about my illness from health facilities which would be transmitted from one to the other*. *They don’t know my illness if I would not tell them or they cannot prevent themselves*. *I think for another person’s health since it would harm them*. *If they know my illness until I cure*, *nothing will have happened to me from anyone*.*”* (20-year-old, male, TB patient)

## Discussion

This study found that there is low household contact tracing and also explored a variety of barriers and facilitators with a wide spectrum of factors related to the health system, health workers, and index TB cases and household contacts and socio-economic and cultural factors that affected TB contact tracing of index TB cases. This study found that of 34 index TB cases household contact registration was done for only 9 (26.5%) index TB cases; totally 37 households. Also, the overall household contact tracing was 43% among all household contacts registered. This finding is low when compared with the findings of studies conducted in Ethiopia at different settings [11, 14]. This difference might happen due to a difference in geographical setup or the nature of the data source [the current study finding data were obtained from the record review].

Long waiting time and limited readiness of health facilities hindered household contacts of TB cases from contact tracing and investigation. This study finding is supported by the study done in Uganda in that long waiting time was mentioned as a barrier for contact tracing which is related to health worker tardiness and absenteeism [15]. Therefore, this underscores the necessity of reducing long waiting time and improving institutional readiness by opening TB clinics on time, improving card room documentation by computerizing, motivating health workers and providing TB training to other health workers to have health workers that will be assigned full-time worker in TB clinics.

Shortage of logistics and infrastructure like unavailability of masks, shortage of reagents, the limited functionality of microscopy, absence of well-ventilated TB class, and interruption of electricity were also found as obstacles that affected effective contact tracing of index TB cases. Interruption of electricity leads appointed for next time and waiting for a long time which affects the clients’ confidence in the health facility. A lack of designated, well-ventilated space for TB-related activities also hindered the ability to provide education and counseling for TB patients and contacts. This finding was supported by studies done in Botswana and Oyo State South West Nigeria where poor or inadequate facilities, poor medical infrastructure, and shortage laboratory diagnostics were mentioned as **barriers to contact tracing service in resource-constrained setting** [16, 17]. Also in contrast to this study finding, a well designated, well-ventilated space is important for health workers to express concerns, discussing sensitive personal health information of patients, and to minimize the time spent with TB patients to reduce the risk of TB transmission [15, 17].

This study found a shortage of human resources at health facilities as a barrier that affected the implementation of TB contact tracing. This finding is in agreement with a study conducted in Canada [18], Thailand and Myanmar [19] as there was often no permanent or full-time nurse or shortage of human resources available in a given community to implement TB programming. Due to a shortage of trained human power, there is incomplete and inadequate contact investigation which leads to missed opportunities to identify secondary active cases and ensure the identification and treatment of infected contacts [9].

Lack of regular monitoring and supervision has given to health centers and health posts were found as a barrier to contact tracing of index TB cases in the current study. This study finding agrees with a study conducted in South Africa that showed a lack of adequate supervision and direction from the district or provincial TB managers [20]. The World Health Organization also recommends conducting routine monitoring using standardized methods based on data with documented quality [5]. Monitoring and supportive supervision can contribute to improved health worker's motivation performance, and fill gaps on time so that they effectively perform their duties [10, 21]. Therefore, strengthening the monitoring and supervision system with a detailed checklist is crucial to achieving the intended goal of contact tracing of index TB cases.

This study indicated that distance influenced household contacts and index cases adherence to contact tracing. Household contacts of TB required going a long distance to reach health facilities for investigation of TB. Due to this, household contacts miss the investigation of TB. This is similar to studies conducted in Brazil and Vietnam which identified the distance between the clinic and their house as a barrier to attend contacting tracing [22, 23].

Socio-economic constraints, such as shortage of money for transportation and laboratory cost were described as barriers for contact tracing. The findings from this study are similar to other studies which found that limited financial resource is a common obstacle to be investigated and traced for TB [12, 16]. Even, when tuberculosis diagnosis and treatment are offered free of charge, social protection measures are needed to alleviate the burden of income loss and non-medical costs of seeking and staying in care [5]. Therefore, this needs the provision of such activities free of charge.

This study identified that poor knowledge and awareness among household contacts and index cases on contact tracing which is similar to study finding conducted in Kenya which showed that TB patients did not know about the need for investigating contacts [24]. This might have negative consequences in that a single person with active TB/ capable of spreading infecting others who remained undetected can infect between ten and fifteen persons every year, making a vicious cycle of failing control efforts [25]. This implies that health education programs are needed to increase awareness on the importance of early contact tracing for household contacts and index cases in the general community by using mass media, community meetings, and conversions. Health workers should counsel and educate to increase awareness of index case and household contacts about TB including the need to have contact tracing.

Another barrier related to a patient in the current study for no-adherence to contact tracing was being busy at work to go health facility. Similarly, the findings of studies conducted in South Africa and Uganda reveal workload was one reason mentioned for TB contacts’ non-attendance of health facilities for evaluation [15, 26].

One problem related to health workers was the lack of motivation or commitment to contact tracing and investigation which affects contact tracing. This is in agreement with the finding of the study conducted in Botswana in that motivation and commitments was mentioned as a barrier for implementation of contact tracing [17]. Health worker's motivation and commitment are also important to deliver better care and to improve the performance of contact tracing [27].

Availability of contact tracing and investigation registration books, referral and screening checklist, and report formats were reported as facilitators for contact tracing. In contrast to this finding, a study conducted in Kenya found that the lack of tools for systematic investigation by health workers, and the lack of documentation tools which were barriers to contact tracing [24] that might happen due to difference in the setup, context, and health system.

Social support like community support and family was identified as facilitators. Study participants mentioned that family support can alleviate economic and social problems, and family and community members are very important to keep them motivated and are sources of encouragement. Similarly, a qualitative study conducted in Uganda [15] found that social support from family was a facilitator for contact tracing.

Socio-cultural factors, such as stigma and discrimination, also had undesirable effects on treatment adherence and early screening of TB adherence and to remaining in care. This study found that there was no stigma and discrimination that index cases and household contacts faced. In contrast to this, studies conducted in Uganda, Vietnam and South Africa showed discrimination and stigma as a major barrier to uptake clinical evaluation at a health facility [15, 26, 28]. The difference might happen from a difference in educational status, socio-cultural differences, lack of SBCC and differences in their understanding and meanings of stigma and discrimination.

## Conclusion

From this study, it was understood that there was a gap in addressing all household contacts which resulted in low household contact tracing of index TB cases. Also, the study explored a wide range of possible barriers and facilitators for it. Explored barriers outweigh the facilitators which might have an implication facilitating the dissemination of TB silently within the community. This underscores the importance of taking action to avert those barriers by developing different strategies to increase TB contact tracing. Therefore, health care providers improve the implementation of contact tracing by designing and developing appropriate strategies that should fit the local context.

## Abbreviations

AFB: Acid Fast Bacilli
HEW: Health Extension Worker
HIV: Human Immune Virus
HMIS: Health Management Information System
IDI: In-depth interview
INH: Isoniazid
MDR: Multidrug resistance
MPH: Master of Public Health
PhD: Philosophical Degree
SBCC: Social and Behavioral Change Communication
SNNPR: Southern Nation and Nationality People’s Representative
TB: Tuberculosis
XDR-TB: Extensively Drug-Resistant Tuberculosis
